# Evolution of the sheep coat: the impact of domestication on its structure and development

**DOI:** 10.1017/S0016672320000063

**Published:** 2020-06-10

**Authors:** Neville Jackson, Ian G. Maddocks, James E. Watts, David Scobie, Rebecca S. Mason, Clare Gordon-Thomson, Sally Stockwell, Geoffrey P.M. Moore

**Affiliations:** 1 CSIRO Division of Animal Production, Prospect, NSW 2148, Australia; 2 PO Box 2604, Bowral, NSW 2576, Australia; 3 AgResearch, Lincoln Research Centre, Private Bag 4749, Christchurch 8140, New Zealand; 4 Discipline of Physiology, School of Medical Sciences, Faculty of Medicine and Health, University of Sydney, Sydney, NSW 2006, Australia; 5 CSIRO Agriculture and Food, Brisbane, Queensland 4067, Australia

**Keywords:** domestication, evolution, follicle morphogenesis, hair, neural crest, sheep, wool

## Abstract

Wild sheep and many primitive domesticated breeds have two coats: coarse hairs covering shorter, finer fibres. Both are shed annually. Exploitation of wool for apparel in the Bronze Age encouraged breeding for denser fleeces and continuously growing white fibres. The Merino is regarded as the culmination of this process. Archaeological discoveries, ancient images and parchment records portray this as an evolutionary progression, spanning millennia. However, examination of the fleeces from feral, two-coated and woolled sheep has revealed a ready facility of the follicle population to change from shedding to continuous growth and to revert from domesticated to primitive states. Modifications to coat structure, colour and composition have occurred in timeframes and to sheep population sizes that exclude the likelihood of variations arising from mutations and natural selection. The features are characteristic of the domestication phenotype: an assemblage of developmental, physiological, skeletal and hormonal modifications common to a wide variety of species under human control. The phenotypic similarities appeared to result from an accumulation of cryptic genetic changes early during vertebrate evolution. Because they did not affect fitness in the wild, the mutations were protected from adverse selection, becoming apparent only after exposure to a domestic environment. The neural crest, a transient embryonic cell population unique to vertebrates, has been implicated in the manifestations of the domesticated phenotype. This hypothesis is discussed with reference to the development of the wool follicle population and the particular roles of Notch pathway genes, culminating in the specific cell interactions that typify follicle initiation.

## 1. Background

Sheep and goats, of the subfamily Caprinae, are amongst the earliest bovids to have been domesticated. Derived from wild species, they inhabited the Fertile Crescent 10,000–12,000 years ago. This region encompassed parts of south-eastern Anatolia, Mesopotamia and western Iran (Reed, 1984; Zeder, 2008). Mitochondrial DNA profiles reveal a number of domestication events (Pedrosa *et al.*, 2005; Pereira *et al.*, 2006; Tapio *et al.*, 2006). Evidence from prehistoric habitation sites (Zeder, 2008) and genetic signatures (Kijas *et al.*, 2012; Fariello *et al.*, 2014) indicates early management and selection strategies, including culling, polling and castration. Although originally from elevated and mountainous terrain, domesticated caprines adapted to a variety of new environments. By tracking retroviral sequences in the ovine genome, Chessa *et al.* (2009) mapped the movements of people and livestock – the so-called ‘Neolithic package’ – to the Far East and westwards to Europe and Africa. The integrations and their predicted mutation rates indicate that present-day European Mouflon, Soay and other North Atlantic island sheep are relics of those early migrations.

Initially sourced for food, archaeological discoveries, contemporary images, parchment inclusions (Ryder & Stephenson, 1968) and other research (Ryder, 1958, 1986; Fraser & Short, 1960; Carter, 1968) indicate that harvesting of coat fibres began some millennia later. Early fleeces were pigmented, with coarse overhairs, similar to those of their wild counterparts. Wool was found in textiles from the early Bronze Age and white wool in the Iron Age (Ryder, 1984; Sherratt, 1984). Fine fibres were identified in parchment made from sheepskin in the Middle East, early in the Modern Era (Ryder, 1958, 1984). Fine wool became a valuable commodity and a symbol of prestige. The pallium, made with white wool, was an exclusive papal vestment in the fourth century (Davis, 1958).

The appearance of domesticated sheep in the Iberian Peninsula has been dated at 5000–7000 BCE (Zeder, 2008; Ciani *et al.*, 2015). Fine-woolled Apulian sheep were introduced during the Roman occupation in the first millennium, and two-coated sheep were introduced from North Africa. Evidence of fine-woolled sheep of Spanish origin emerged around the thirteenth century. The Merino was possibly the first recognized breed in the late Middle Ages, spreading through Europe, Asia and Australia during the eighteenth and nineteenth centuries (Carter & Clarke, 1957b; Ryder, 1964; Ciani *et al.*, 2015). Table 1 summarizes changes in fleece traits following domestication (Carter, 1955; Ryder, 1958, 1960, 1964, 1966, 1986; Ryder & Stephenson, 1968; Sherratt, 1984).

**Table 1.** Approximate phases for the appearance of changes in sheep types and fleece and fibre traits from wild sheep to the Merino.

Evolution	Timescale	Sheep, skin or fibre samples	Primary fibre diameter (Dp, μm)	Secondary fibre diameter (Ds, μm)	Fibre medullation	Secondary/primary follicle ratio (S/P)	Fleece structure
Wild sheep	10,000 BCE	Mouflon, Argali, Urial	150	15	Latticed	3–5	Two coated: long medullated overhairs, fine underwool
Primitive domestic sheep	7000–5000 BCE	Soay, Asiatic	42	17	Non-latticed, continuous	4–5	Ill-defined staples, fine fibres
Ancient fine/medium wool	500 BCE	Dead Sea scrolls; ancient textiles	38	21	Discontinuous	5–7	Coarse overhairs, fine–medium fibres
Fine wool	1500–1800	Spanish Merino	19–24	17–21	Absent	20	Well-defined staples, fibres largely uniform in diameter and length
Australian Merino Fine Medium Strong	1830–present		16–22 21–29 29–32	16–21 16–24 22–25	– – –	16–24 19–27 15–18	Well-defined staples, fibres uniformly fine–medium diameter, uniform length

## 2. Follicle and fibre types

The hair coats of wild and many domesticated caprines are similar in appearance and structure (Ryder, 1958). Commonly pigmented brown or black, they are composed of a layer of long, coarse, medullated primary (P) fibres of variable diameters. These cover an undercoat of shorter, finer, unmedullated secondary (S) fibres. Coarse fibres are three to five times the diameter of fine fibres, with densities between 3 and 5/mm^2^, secondary/primary (S/P) follicle ratios of 3–4 (Carter, 1968) and fleece weights of about 1 kg. There are annual cycles of growth and shedding (Carter, 1955; Ryder & Stephenson, 1968). Here, for convenience, these traits will be referred to as ‘primitive’.

**Fig. 1.**
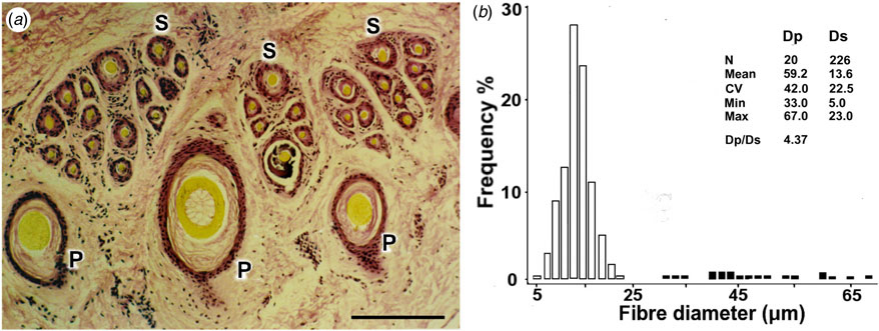
Transverse skin section and fibre diameter distribution of Barbary sheep. (a) Primary (P) follicles in a trio group; fibres are large, medullated and of variable diameters (P Ø: 32, 53 and 26 μm, respectively). Secondary (S) follicles are in wedge-shaped clusters, adjacent to P follicles and have finer, shorter fibres (S Ø: 9 μm). Bar = 100 μm. (b) Histogram of the distribution of P and S fibre diameters (Dp, Ds) in the skin sample. P fibre frequencies are shaded.

A micrograph of a skin section from a two-coated Barbary sheep (Ryder, 1958; Mason, 1967) is depicted in Fig. 1a, showing a trio group of coarse P fibres adjacent to clusters of fine S fibres. The fibre diameter distributions are plotted in the histogram of Fig. 1b.

**Fig. 2.**
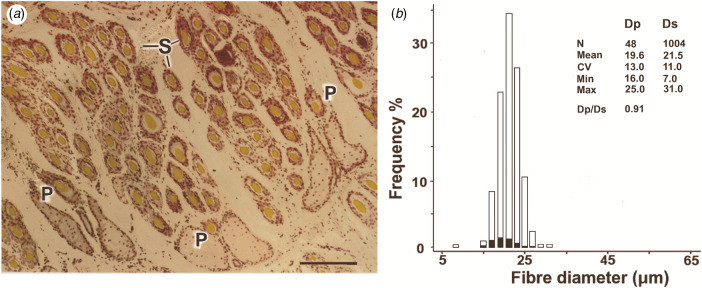
Transverse skin section and fibre diameter distribution of a modern Merino sheep. (a) Primary (P) follicles in a trio group; P fibres are fine, not medullated and have similar diameters (Ø: 20 μm). Secondary (S) follicles are numerous and are also of uniform diameter (Ø: 22 μm). Bar = 100 μm. (b) Histogram of the distribution of P and S fibre diameters (Dp, Ds) in the skin sample. P fibre frequencies are shaded.

By contrast, modern (hereafter: ‘woolled’) sheep, used for the manufacture of textiles, have fleeces composed of predominantly white, unmedullated P and S fibres, S/P ratios of 5–20 and wool weights of 3–5 kg (Carter & Clarke, 1957a; Carter, 1968). Wool growth is continuous and fibre diameters are more uniform, with P/S fibre diameter (Dp/Ds) ratios ranging from 1.0 to 1.3 (Carter, 1968). Figure 2a depicts a **t**ransverse skin section from a fine Merino, with Fig. 2b showing a histogram of the fibre diameters. The P and S distributions are superimposed.

**Fig. 3.**
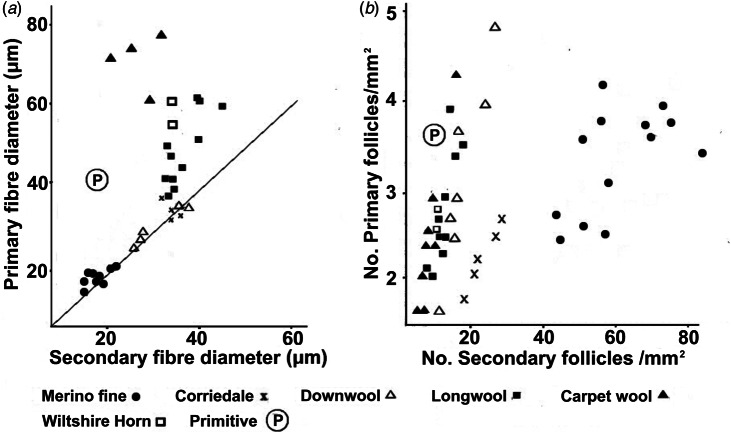
Comparisons of (a) primary and secondary fibre diameters and (b) primary and secondary follicle densities in a range of modern breeds; the primitive Soay sheep is designated ℗. Data from Carter (1968). Corriedales (Merino × Longwool) display intermediate characteristics. A primary to secondary fibre diameter ratio of 1 is shown as a diagonal line in (a).

Comparisons between woolled and primitive breeds show the extent of the differences in P and S fibre diameters and follicle densities (Figs 3a & 3b). Data from Merino sheep fall into clusters, separated from both primitive sheep and other modern English breeds.

Carter (1965) commented that secondary follicle density in the Merino could be an order of magnitude greater and fleece weights three to five times those of other modern breeds.

## 3. Evolution of the coat

The prevailing view of the evolution of modern sheep is from an archaeological perspective (Ryder, 1964), with a millennial timescale (Table 1). It was postulated that conversion from a hairy two-coated animal with an annual moult to one with continuously growing fibres occurred via an intermediate stage, typified by the ‘woolly Soay’ of the Outer Hebrides. Coarse hairs were gradually replaced with finer, continuously growing fibres and fibre diameters transitioned from a skewed, fine-to-coarse profile to a normal distribution, as illustrated in Fig. 2b. Earlier, Fraser (1953) proposed an evolutionary path with no intermediate stages, but this was highly speculative, involving a ‘fibre substrate’. Despite this, the direct path has received support from recent genotype analyses. Employing models that measured gene flow between sheep breeds, Ciani *et al.* (2015) showed the highest values amongst early Merino and wild/feral sheep. Indeed, the studies suggest not only that differences between primitive and woolled animals were small, but that changes from one to the other were achieved, or were achievable, rapidly. The implications of these observations are that the Merino fleece may not be as remote from its two-coated origins as generally assumed.

To explore this, we have re-examined the nature of the fleece with the benefit of more recent insights into factors affecting the development of the follicle population and new perspectives on the consequences of domestication.

## 4. Development of the follicle population

The basic structure and composition of the coats and the development and distribution of the follicle populations are alike in primitive and woolled sheep. Follicles are initiated in overlapping waves and at similar times, during foetal life (Fraser, 1953; Marston, 1955). The P follicles are formed first, commonly arranged in trio groups. Densities average 3–5/mm^2^, and each is associated with a sweat gland and arrector muscle (Tänzer, 1926; Carter, 1968; Tuncer *et al.*, 2018). Later, S follicles develop, initially at non-randomly spaced sites in the skin: the secondary original follicles. Finally, secondary derived follicles arise by branching from other S follicles (Hardy & Lyne, 1956). Derived follicles are highly variable in number and are primarily responsible for differences in follicle densities amongst breeds (Carter, 1968; Moore *et al.*, 1998). They are also widespread, having been reported both in woolled (Carter & Clarke, 1957a, 1957b; Orwin, 1961) and primitive sheep, including the Karakul (Tänzer, 1926) and Soay (Ryder, 1959).

## 5. Fibre shedding

Two-coated sheep undergo annual cycles of growth and shedding. Ryder (1960, 1966) described a spring moult in the Soay and European Mouflon and Slee (1965) in the Wiltshire Horn. Ryder (1962) reported shedding in Merinos, but this was not confirmed (Lyne, 1961; Ryder, 1967). It is generally accepted that the fleece grows continuously and that moulting, if it occurs, is negligible. However, Merinos show annual rhythms of wool growth that coincide with those observed in shedding sheep (Bennett *et al.*, 1962; Hutchinson, 1965). These are more pronounced in haired regions, perhaps echoing an archaic hair growth cycle.

A significant insight into a means by which continuous wool growth might have superseded shedding was reported by O'Connell *et al.* (2012). Selective breeding programmes to increase and decrease greasy fleece weights (up- and down-selection) in Wiltshire Horn sheep revealed an inverse correlation between fleece weight and the extent of shedding over the body. The transformation from two coats to continuous growth occurred progressively, encompassing 80% of the fleece after 8 years of selection.

## 6. Primitive traits in Merinos

Sheep with primitive traits appear sporadically but persistently in research and industry Merino flocks in Australia. These include, variously, individuals with coarse, occasionally medullated P fibres, high Dp/Ds ratios and low follicle densities. Although mostly anecdotal, there are some published reports. Cox (1936) described a Merino ram with “extraordinary wool growth, which is said to be similar to the original wild sheep, having a coating of short wool and hair.” Marston (1955) noted that some Merino strains grew long, coarse fibres resembling those of the outer coats of primitive sheep. Similarly, a medium Merino flock with a mean S/P ratio of 22 and a follicle density of 64/mm^2^ included a ewe with an S/P ratio of 3–5 and a follicle density of 20/mm^2^ (Carter & Clarke, 1957a).

Coarse fibres have been found in individual animals from both stud and research flocks. Using Dp/Ds values as a comparative measure of fibre diameter distributions (Carter & Clarke, 1957a), ratios >1.5 were found in ewes in almost all strains and environments (Gallagher, 1970; Gallagher & Yeates, 1970).

**Fig. 4.**
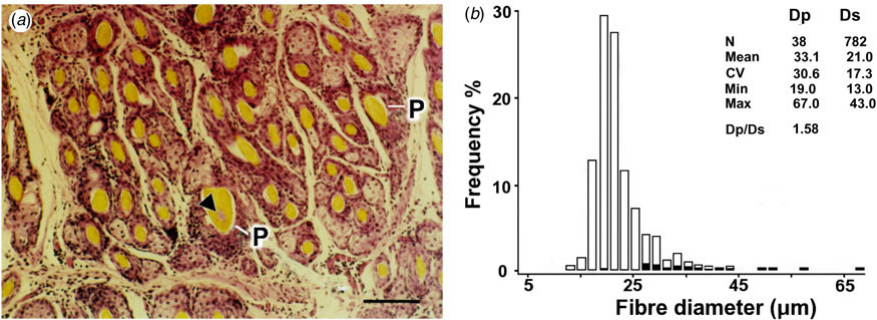
Transverse skin section and primary (P) and secondary (S) fibre diameter distribution of a ewe from the follicle size and number selection experiment. (a) The fibre of the P follicle (P at centre) is large and medullated (arrowhead); also shown is an adjacent P follicle with an unmedullated fibre. Bar = 100 μm. (b) Fibre diameter histogram from the same skin sample, showing P and S fibre diameter (Dp, Ds) distributions. Ds values range from coarse to fine. P frequencies are shaded.

Further, an up- and down-selection programme to determine the effects of changes in follicle size and number on wool growth, which ran for 8 years (Jackson *et al.*, 1979), generated a number of animals with primitive characteristics. Figures 4a and 4b depict a skin section and fibre diameter distribution of a high selection line ewe at the end of the experiment. Large medullated P fibres were present and P and S diameter distributions were skewed into the coarse range.

## 7. Feral sheep

Although human-mediated selection has not focused primarily on environmental fitness, there are many instances of domesticates escaping to, and thriving, in ecosystems unlike those of their wild origins. Whilst the Soay and European Mouflon are probably the best known, their returns to the wild occurred before coat fibres had become commodified (Poplin, 1979). Feral Merino populations are, by contrast, more recent phenomena, having been reported on islands in Hawaii, California, the Solomons and New Zealand (Orwin & Whitaker, 1984; Sumner *et al.*, 2017). The provenance of most flocks is not well documented. However, an isolated population on Arapawa Island, New Zealand, appear to have originated from Merino breeds (Orwin & Whitaker, 1984; Pickering *et al.*, 2013; Ciani *et al.*, 2015). Reportedly sourced from Australia in the nineteenth century, animals were abandoned or escaped in the 1860s, eventually consolidating into a fairly stable population of small flocks. A detailed study in the 1980s found that many of the traits exhibited by the animals were those of primitive breeds. They were observed to lose their fleeces, beginning with belly and neck regions. Shedding was achieved by a combination of seasonal fibre thinning and physical abrasion, a behaviour observed in the Mouflon (Ryder, 1960). Coats were commonly pigmented and fleece weights were about half those of mid-nineteenth-century Merinos (Massy, 2007). Mean fibre diameter (23.1 μm) was similar to that of a medium-woolled Merino, but diameter distributions were positively skewed, ranging from 9 to 109 μm. Some larger fibres were medullated. The skin had a follicle density of 26.9/mm^2^ and an S/P ratio of 6.0 (Orwin & Whitaker, 1984).

**Fig. 5.**
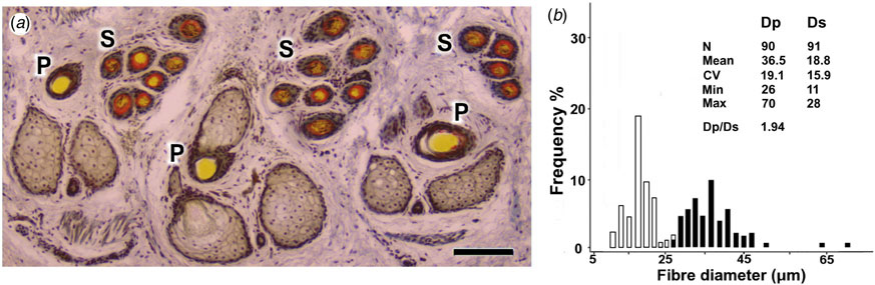
Transverse skin section and fibre diameter distribution of Arapawa Island sheep. (a) Trio group of primary (P) follicles. Secondary (S) follicles located are in wedge-shaped clusters between the P follicles. Bar = 100 μm. (b) Fibre diameter histogram from the same skin samples, showing P and S fibre distributions (Dp, Ds). P fibre frequencies are shaded.

A transverse skin section and a histogram of fibre diameters from three Arapawa ewes sampled in 1993–1994 are shown in Figures 5a and 5b. The P and S fibre diameters fall into two distributions with means of 37 and 19 μm, respectively. They approximate those of the 1985 high selection line Merino of Fig. 4b.

**Fig. 6.**
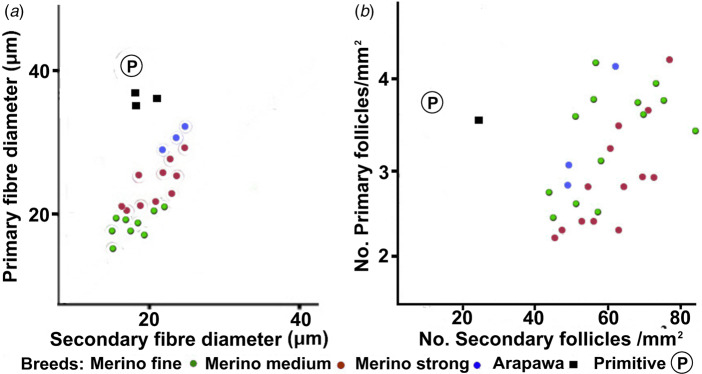
Comparisons between (a) primary and secondary fibre diameters and (b) primary and secondary follicle densities in modern Merino breeds (Carter, 1968) and in Arapawa sheep. The Arapawa mean fibre diameters in (a) are from the data of Fig. 5b. The follicle density measurement in (b) is from Orwin & Whitaker (1984); the Soay sheep ℗ and Merino values are from Carter (1968).

Comparisons between modern Merino P and S diameters and densities and those of primitive (Soay) and Arapawa sheep are depicted in Figs 6a and 6b.

The transformations observed in this small, isolated sheep population occurred within approximately 30 generations, indicating that adaptation to the prevailing environment was not only rapid, but occurred in a sufficiently large proportion of the animals to ensure survival. The changes in fleece traits are consistent with the observations of Darwin (1868), who noted the rapidity with which selective breeding could generate new types, the difference being that the changes in Arapawa sheep were a reversion, having occurred in the absence of human intervention. Given the small size of the population, the likelihood that the animals evolved as a consequence of random mutations coupled with natural selection is remote.

## 8. The domesticated phenotype

One aspect of the evolution of the fleece not previously addressed has been the effect of the domestication process itself, beginning at the capture of animals from the wild and introducing them to an environment created by humans. The pioneering work of the Soviet scientist Dmitry Belayev, using wild silver foxes, demonstrated that by selecting individuals for a single behavioural trait (tameness), an array of seemingly unrelated developmental, physiological, hormonal and skeletal modifications were induced (Belayev, 1969, 1979). In addition to a progressively increasing tolerance of human proximity, there were alterations to the shape and size of the skull and long bones, brain size was reduced and the breeding season was prolonged. Tameness in young foxes was correlated with reductions in corticotrophin-releasing hormone secretion, adrenal gland size and circulating cortisol levels (Trut *et al.*, 2009). Coat pigmentation altered, blazes and white areas developed and there were changes in hair length, texture and seasonal moulting. These appeared rapidly and more or less concurrently. More than half of the foxes in the experiment exhibited the traits after 50 years of selection.

Perhaps more remarkably, the effects of domestication were not confined to silver foxes. Similar arrays of physical and behavioural traits were reportedly induced in otherwise unrelated vertebrates (sheep, horses, dogs, etc.) in which the only common denominator was that of being brought under human control (Trut *et al.*, 2009; Wilkins *et al.*, 2014). The term ‘domestication syndrome’ was abstracted by Wilkins *et al.* (2014) to describe the suite of traits recurring in species in which evolutionary pathways did not cross but had nevertheless evolved in the same direction.

## 9. The neural crest

The genetic and developmental mechanisms underlying domestication have been widely canvassed. The observations suggested that mutations with equivalent effects were present in the same genes. The phenotypic similarities in such a range of species seemed likely to result from a gradual accumulation of cryptic genetic changes early during vertebrate evolution. The implications are that the mutations, because they did not affect fitness in the wild, were not exposed to adverse selection. Their frequencies were therefore inherited unchanged, becoming increasingly buffered against alterations as species diverged. Their accumulated effects only became apparent following exposure to a human environment.

Wilkins *et al.* (2014) postulated the involvement of the neural crest (NC), a transient embryonic cell population unique to vertebrates and of considerable antiquity (Sauka-Spengler *et al.*, 2007). Differentiating early during embryogenesis, it gives rise to a population of multipotent, self-renewing NC cells (NCCs) with extensive developmental and regulatory functions. Following an epithelial to mesenchymal transition (Simoes-Costa & Bronner, 2013), NCCs migrate extensively and contribute to the specification of many tissues that characterize the domesticated phenotype, differentiating into pigment cells, elements of the nervous system, musculature, connective tissue, cartilage, bone and skin (Le Douarin, 1968; Adameyko *et al.*, 2009).

It was proposed that the domesticated phenotype developed as a consequence of deficits in proliferation or migratory capacities of the NCCs, resulting in lower densities at their final destinations (Wilkins *et al.*, 2014). Whilst consistent with alterations in some physical features and perhaps pigmentation, changes in hair texture and increases in fibre growth and follicle density suggest an alternative explanation: that of redirecting NCCs to alternative developmental pathways (Kulesa *et al.*, 2000). Clonal analysis has confirmed that single NCCs can not only differentiate into a variety of cell types, but also propagate cells with similar potential (Bronner-Fraser & Fraser, 1988). Thus, for example, whilst differentiating into pigment cells in skin (Le Douarin, 1968), melanoblasts may also generate sub-lineages with functions not involving melanin synthesis (Aoki *et al.*, 2009; Uehara *et al.*, 2009).

## 10. Neural crest cells and follicle morphogenesis

We propose that, in the sheep, rather than failing to migrate, NCCs are redirected to fates that include an increased commitment to hair follicle morphogenesis. There is evidence to support this view. NCCs are found in epidermal and dermal compartments of the skin and hair follicles, together with NC-derived cells (Fernandes *et al.*, 2004) and those expressing NCC markers. The origins and fates of NCCs and NC-derived cells have been actively debated (Sieber-Blum *et al.*, 2004; Jinno *et al.*, 2010). However, attenuation of Wnt signalling using Cre/LoxP-knockout technology (Li *et al.*, 2007) and, more specifically, in NCCs (Narytnyk *et al.*, 2014) resulted in reductions in both hair follicle density and fibre diameter, implicating NCCs in that specific morphogenetic process.

Previously, we have shown that both follicle density and fibre diameter in the sheep are determined at follicle initiation (Moore *et al.*, 1989, 1996). Each follicle and the dimensions of its fibre are specified by the numbers of mesenchymal cells that condense into a papilla at an initiation site (Moore *et al.*, 1998). Prepapilla cells are derived from a lineage that differentiates in the mesenchyme at follicle initiation. They participate in the development of the whole follicle population (Moore *et al.*, 1996, 1998). At initiation, the cells aggregate as a consequence of transient Notch signalling (Gordon-Thomson *et al.*, 2008) and intercellular Notch–Delta and Delta–Delta interactions (Xavier *et al.*, 2013). In association with an epidermal cell cluster, they constitute a follicle primordium at each initiation site. The reductions in follicle size and density that result from NCC inactivation (Narytnyk *et al.*, 2014) and the presence of NCCs in follicle papillae (Fernandes *et al.*, 2004; Sieber-Blum *et al.*, 2004) place NCCs in the papilla aggregates at follicle initiation.

## 11. Conclusions

Primitive sheep and the modern Merino are generally regarded as occupying either end of a range of fibre-producing breeds. However, examination of the nature of the fleece and its ready adaptability suggest that the differences are less striking than their similarities. The development and types of follicles initiated are essentially identical, and similar growth cycles are present, albeit to varying degrees. The rapidity and apparent facility with which a follicle population may convert from shedding to continuous growth and revert from domesticated to feral states argue that the apparent differences are simply stages in a spectrum of types, ranging from coarse fibres and two coats to fine and continuously growing. The random appearance of primitive traits in modern flocks then becomes a not unexpected occurrence. A labile process appears to be in play.

Domestication has emerged as a dynamic feature of the evolution of sheep breeds. Within this environment, modifications to the coat structure and composition have occurred in timeframes and to sheep population sizes that exclude evolutionary variations arising from random mutations and natural selection. The fact that these changes occur so consistently and, from available evidence, rapidly, points to an accumulation of a suite of genetic changes that only achieved phenotypic expression through exposure to an environment managed by humans. The involvement of NCCs in follicle morphogenesis is a working hypothesis that provides a single, coherent and essentially consistent rationale for the appearance of changes in coat structure, characteristics and composition associated with a domesticated phenotype.

Seemingly, the Merino has access to an extensive wardrobe. What it is wearing at any particular time is a consequence of two competing and unrelenting environmental forces: one natural, the other human.

## Materials and methods

Skin preparation

Skin samples were harvested from the mid-side of each animal using a 10 mm Ø trephine, fixed and stained using standard histological procedures, but omitting haematoxylin staining (Maddocks & Jackson, 1988).

Automated measurement of follicle and fibre characters

Data pertaining to follicle and fibre characters were collected from skin samples by standard microscopic examination of histological material or automated imaging. For the latter procedure, sectioned material was examined on an image analysis system consisting of a Kontron IBAS 2 image-processing computer and a Zeiss Research microscope equipped with a high-resolution monochrome video camera and a Zeiss monochromator. The system scanned as many microscope fields as could be obtained from the specimen, moving in a pattern that avoided resampling of areas already measured. For each field, fibre images were segmented and P fibres identified.

P and S fibres were counted and measured separately (Carter, 1943). Results were presented as P, S and total follicle counts/mm^2^ of skin, S/P, mean diameter, standard deviation and coefficient of variation for P, S and total fibre numbers, respectively. Data from each skin sample were prepared as a histogram of fibre diameters as frequencies in 32 × 2 μm classes.
